# Supplementary material for: Artificial Intelligence and Machine Learning Applications to Pharmacokinetic Modeling and Dose Prediction of Antibiotics: A Scoping Review
Source: Antibiotics (Basel). 2024 Dec 10;13(12):1203. doi: 10.3390/antibiotics13121203 (PMC11672403; doi:10.3390/antibiotics13121203)
Supplement: Supplementary file 1 [file antibiotics-13-01203-s001.zip › antibiotics-3335544-supplementary.pdf]

Supplementary Information

Artificial Intelligence and Machine Learning techniques

Naive Bayes

The Naive Bayes classifier is one of the simplest and most effective classification algorithms that help create fast ML models that can make quick predictions. It is mainly used for text classification of high-dimensional training datasets. The most common applications are text classification, such as medical data, spam filtering, or sentiment analysis *[1]*.

Decision Trees (DTs)

Decision Trees (DTs) are non-parametric algorithms used for classification and regression. It can solve problems for both categorical and numerical data. Their goal is to build a model that predicts the value of a target variable by learning simple decision rules derived from data features. It has a hierarchical tree structure consisting of root nodes, branches, internal nodes, and leaf nodes. Each internal node represents the "test" for an attribute, each branch represents the result of the test, and each leaf node represents the final decision or result *[2–4]*.

Support Vector Machines (SVMs)

Support vector machines (SVMs) combine techniques that can be used for classification, regression, and outlier detection. Binary or multiple classifications can be implemented. Its main purpose is to be effective in large spaces, even if there are more of them than samples.

The goal of the SVM algorithm is to create the best line or decision boundary that can divide the n-dimensional space into classes so that we can easily classify new data points into the correct categories in the future. This optimal decision boundary is called a hyperplane. The SVM selects extreme points/vectors that help create a hyperplane. These extreme cases are called support vectors, so the algorithm is called a support vector machine *[5,6]*.

K-Nearest Neighbors (KNNs)

The K-Nearest Neighbors (KNNs) algorithm is a general and widely used ML algorithm that is mainly used for its simplicity and ease of implementation. No assumptions are required about the underlying data distribution. It can handle both numerical and categorical data, making it a flexible choice for different types of datasets in classification and regression tasks. It is a non-parametric method that makes predictions based on the similarity of data points in a given dataset. Compared to other algorithms, K-NN is less sensitive to outliers. The K-NN algorithm works by determining the K-nearest neighbors of a given data point. The class or value of a data point is then determined by a majority vote or the average of K neighbors. This approach allows the algorithm to adapt to different patterns and make predictions based on the local structure of the data *[7,8]*.

Linear Regression

Linear regression is a type of supervised ML algorithm that computes the linear relationship between a dependent variable and one or more independent features. When the number of the independent features is "1", then it is known as univariate linear regression, and in the case of more than one feature, it is known as multivariate linear regression *[9]*.

Logistic Regression

Logistic regression is another supervised learning algorithm that is used to solve classification problems. In classification problems, we have dependent variables in a binary or discrete format such as "0" or "1". The logistic regression algorithm works with categorical variables such as "0"

or "1", "Yes" or "No", etc. Logistic regression uses a sigmoid function or logistic function which is a complex cost function *[9,10]*.

Polynomial Regression

Polynomial regression is a type of regression that uses linear models to model nonlinear datasets. It is similar to multivariate linear regression but fits a nonlinear curve between the value of "x" and the corresponding conditional value of "y"

Suppose there is a dataset that consists of data points that are present in a nonlinear fashion; for such a case, linear regression will not fit those data points the best. To cover such data points, we need polynomial regression.

In polynomial regression, the original features are transformed into polynomial features of a given degree and then modeled using a linear model. This means the data points are best fitted using a polynomial line *[9]*.

Ridge Regression

Ridge regression is a regularization technique, which is used to reduce the complexity of the model; it is also called L2 regularization. It is one of the most robust versions of linear regression in which a small amount of bias is introduced so that we can obtain better long-term predictions. The amount of bias added to the model is known as the ridge regression penalty. We can compute this penalty term by multiplying lambda by the squared weight of each individual feature *[11,12]*.

Lasso Regression

Lasso regression is another regularization technique to reduce the complexity of the model and it is also called L1 regularization. It is similar to the ridge regression except that the penalty term contains only the absolute weights instead of a square of weights *[13]*.

Elastic Net (EN)

Elastic Net (EN) is another method that linearly combines the penalties of ridge and LASSO. Compared to LASSO, EN can generate a model that contains more features than observations. Furthermore, EN exhibits grouping effects, where highly correlated features have similar estimated coefficients [14].

Bagging

Bagging, also known as bootstrap aggregating, is an ensemble learning technique that helps to improve the performance and accuracy of ML algorithms. It is used to deal with bias–variance trade-offs and reduces the variance of a prediction model. Bagging avoids overfitting of data and is used for both regression and classification models, specifically for decision tree (DT) algorithms. Bagging is used when our objective is to reduce the variance of a decision tree. Here, the concept is to create a few subsets of data from the training sample, which is chosen randomly with replacement. Then, each collection of subset data is used to prepare their decision trees; thus, we end up with an ensemble of various models. The average of all the assumptions from numerous trees is used, which is more powerful than a single decision tree.

Boosting

Boosting is another ensemble procedure to make a collection of predictors. In other words, we fit consecutive trees, usually random samples, and at each step, the objective is to solve net error from the prior trees. If a given input is misclassified by theory, then its weight is increased so that the upcoming hypothesis is more likely to classify it correctly by consolidating the entire set and finally converting weak learners into better-performing models *[15]*.

Gradient Boosting

Gradient boosting is an expansion of the boosting procedure. It is a popular supervised ML technique that aggregates an ensemble of weak individual models to obtain a more accurate final model. Gradient boosting is a unique ensemble method since it involves identifying the shortcomings of weak models and incrementally or sequentially building a final ensemble model using a loss function that is optimized with gradient descent. Decision trees are typically the weak learners in gradient boosting, and consequently, the technique is sometimes referred to as Gradient Tree Boosting *[15,16]*.

eXtreme Gradient Boosting (XGBoost)

eXtreme Gradient Boosting (XGBoost) is also a boosting ML algorithm, which is the next version on top of the gradient boosting algorithm. The full name of the XGBoost algorithm is, as the name suggests, an extreme version of the previous gradient boosting algorithm.

The main difference between gradient boosting and XGBoost is that XGboost uses a regularization technique. In simple words, it is a regularized form of the existing gradient boosting algorithm. Due to this, XGBoost performs better than a normal gradient boosting algorithm and that is why it is much faster. It also performs better when there is a presence of numerical and categorical features in the dataset *[17]*.

CatBoost

CatBoost uses decision trees for classification and regression. It has two main entities: it works with categorical data and it uses gradient enhancement. It overcomes the limitation of other decision tree-based methods in which, in general, data must be preprocessed to convert categorical string variables to numerical values, one-hot coding, and so on. This method can directly consume a combination of categorical and non-categorical explanatory variables without preprocessing. CatBoost uses a method called ordered coding to code category entities, which considers the target statistics of all rows preceding a data point to calculate a value to replace the categorical entity. Another unique feature of CatBoost is that it uses symmetric trees, this means that at each depth level, all decision nodes use the same split condition. CatBoost can also be faster than other methods such as XGBoost *[18,19]*.

LightGBM

LightGBM is a gradient boosting ensemble method based on decision trees. As with other decision tree-based methods, LightGBM can be used for both classification and regression. LightGBM is optimized for high performance with distributed systems. LightGBM creates decision trees that grow by leaves, meaning that, given a condition, only a single leaf is split, depending on the gain. Sometimes leaf trees can be overfitting, especially with smaller datasets *[20]*.

Adaptive Boosting (Adaboost)

Adaptive Boosting (AdaBoost) is a supervised ML algorithm used to improve the accuracy of weak classification models. The AdaBoost algorithm iteratively trains a series of weak classifiers on different subsets of data and assigns higher weights to data that were misclassified in previous iterations. The results of these weak classifiers are then combined into a weighted strong classifier, where the better-performing weak classifiers have higher weights in the final classification. The AdaBoost algorithm is known for its ability to significantly improve the accuracy in complex classification tasks with large and noisy datasets. In addition, it is easy to implement and can adapt to various weak algorithms, making it popular in ML practice *[21]*.

Random Forest (RF)

Random Forest (RF) is one of the most powerful supervised learning algorithms that can perform regression as well as classification tasks. It uses the Bagging technique of ensemble learning in

which aggregated decision trees run in parallel and do not interact with each other. RF regression is an ensemble learning method that combines multiple decision trees and predicts the final output based on the average of each tree's output *[22]*.

Extra Trees (ETs)

Extra Trees (ETs) are a version of the RF. Some differences are that RF uses bootstrap replicas whereas ETs use the whole original sample and that RF chooses the optimum split while ETs choose it randomly. However, once the split points are selected, the two algorithms choose the best one between all the subsets of features. These differences motivate the reduction in both bias and variance. On one hand, using the whole original sample instead of a bootstrap replica will reduce bias. On the other hand, randomly choosing the split point of each node will reduce the variance *[23]*.

Neural Networks (NNs)

Neural networks (NNs) are one of the deep learning algorithms that simulate the workings of neurons in the human brain.

The main difference between NNs and linear regression is that linear regression can only learn the linear relationship between the features and target while NNs can learn the complex nonlinear relationship. NNs have the ability to learn the complex relationship between the features and target due to the presence of an activation function in each layer. NNs consist of the input layer, hidden layers, and output layer. The hidden layer can be more than one in number. Each layer consists of an "n" number of neurons. Each layer has an activation function associated with each of the neurons. The activation function is the function that is responsible for introducing nonlinearity in the relationship *[24,25]*.

Multilayer Perceptron (MLP)

A multilayer perceptron (MLP) is a misnomer for a modern feedforward artificial neural networks, consisting of fully connected neurons with a nonlinear kind of activation function, organized in at least three layers, and notable for being able to distinguish data that are not linearly separable. It is a misnomer because the original perceptron used a Heaviside step function instead of a nonlinear kind of activation function *[26,27]*.

Bibliography

1. Lewis, D.D. Naive (Bayes) at Forty: The Independence Assumption in Information Retrieval.; Springer Berlin Heidelberg: Berlin, Heidelberg, 1998; pp. 4–15.
2. Podgorelec, V.; Kokol, P.; Stiglic, B.; Rozman, I. Decision Trees: An Overview and Their Use in Medicine. *J Med Syst* **2002**, *26*, 445–463, doi:10.1023/a:1016409317640.
3. Classification and Regression Trees | Leo Breiman | Taylor & Francis e Available online: https://www.taylorfrancis.com/books/mono/10.1201/9781315139470/classification-regression-trees-leo-breiman (accessed on 10 January 2024).
4. Quinlan, J.R. Induction of Decision Trees. *Machine learning* **1986**, *1*, 81–106.
5. Cortes, C.; Vapnik, V. Support-Vector Networks. *Machine learning* **1995**, *20*, 273–297.
6. Suthaharan, S. Machine Learning Models and Algorithms for Big Data Classification. *Integr. Ser. Inf. Syst* **2016**, *36*, 1–12.
7. Abu Alfeilat, H.A.; Hassanat, A.B.A.; Lasassmeh, O.; Tarawneh, A.S.; Alhasanat, M.B.; Eyal Salman, H.S.; Prasath, V.B.S. Effects of Distance Measure Choice on K-Nearest Neighbor Classifier Performance: A Review. *Big Data* **2019**, *7*, 221–248, doi:10.1089/big.2018.0175.

8. Cover, T.; Hart, P. Nearest Neighbor Pattern Classification. *IEEE transactions on information theory* **1967**, *13*, 21–27.
9. Talevi, A.; Morales, J.F.; Hather, G.; Podichetty, J.T.; Kim, S.; Bloomingdale, P.C.; Kim, S.; Burton, J.; Brown, J.D.; Winterstein, A.G.; et al. Machine Learning in Drug Discovery and Development Part 1: A Primer. *CPT: Pharmacometrics & Systems Pharmacology* **2020**, *9*, 129–142, doi:10.1002/psp4.12491.
10. Hastie, T.; Tibshirani, R.; Friedman, J.H.; Friedman, J.H. *The Elements of Statistical Learning: Data Mining, Inference, and Prediction*; Springer, 2009; Vol. 2;.
11. Hoerl, A.E.; Kennard, R.W. Ridge Regression: Biased Estimation for Nonorthogonal Problems. *Technometrics* **1970**, *12*, 55–67, doi:10.2307/1267351.
12. Hoerl, A.E.; Kennard, R.W. Ridge Regression: Applications to Nonorthogonal Problems. *Technometrics* **1970**, *12*, 69–82, doi:10.2307/1267352.
13. Tibshirani, R. Regression Shrinkage and Selection via the Lasso. *Journal of the Royal Statistical Society. Series B (Methodological)* **1996**, *58*, 267–288.
14. Zou, H.; Hastie, T. Regularization and Variable Selection via the Elastic Net. *Journal of the Royal Statistical Society. Series B (Statistical Methodology)* **2005**, *67*, 301–320.
15. James, G.; Witten, D.; Hastie, T.; Tibshirani, R. *An Introduction to Statistical Learning*; Springer, 2013; Vol. 112;.
16. Friedman, J.H. Greedy Function Approximation: A Gradient Boosting Machine. *Annals of statistics* **2001**, 1189–1232.
17. Chen, T.; He, T.; Benesty, M.; Khotilovich, V.; Tang, Y.; Cho, H.; Chen, K.; Mitchell, R.; Cano, I.; Zhou, T. Xgboost: Extreme Gradient Boosting. *R package version 0.4-2* **2015**, *1*, 1–4.
18. Dorogush, A.V.; Ershov, V.; Gulin, A. CatBoost: Gradient Boosting with Categorical Features Support. *arXiv preprint arXiv:1810.11363* **2018**.
19. Hancock, J.T.; Khoshgoftaar, T.M. CatBoost for Big Data: An Interdisciplinary Review. *J Big Data* **2020**, *7*, 94, doi:10.1186/s40537-020-00369-8.
20. Sheridan, R.P.; Liaw, A.; Tudor, M. Light Gradient Boosting Machine as a Regression Method for Quantitative Structure-Activity Relationships. *arXiv preprint arXiv:2105.08626* **2021**.
21. Hatwell, J.; Gaber, M.M.; Atif Azad, R.M. Ada-WHIPS: Explaining AdaBoost Classification with Applications in the Health Sciences. *BMC Med Inform Decis Mak* **2020**, *20*, 250, doi:10.1186/s12911-020-01201-2.
22. Breiman, L. Random Forests. *Machine learning* **2001**, *45*, 5–32.
23. Geurts, P.; Ernst, D.; Wehenkel, L. Extremely Randomized Trees. *Machine learning* **2006**, *63*, 3–42.
24. Ma, J.; Sheridan, R.P.; Liaw, A.; Dahl, G.E.; Svetnik, V. Deep Neural Nets as a Method for Quantitative Structure–Activity Relationships. *Journal of chemical information and modeling* **2015**, *55*, 263–274.
25. Hinton, G.; Deng, L.; Yu, D.; Dahl, G.E.; Mohamed, A.; Jaitly, N.; Senior, A.; Vanhoucke, V.; Nguyen, P.; Sainath, T.N. Deep Neural Networks for Acoustic Modeling in Speech Recognition: The Shared Views of Four Research Groups. *IEEE Signal processing magazine* **2012**, *29*, 82–97.
26. Popescu, M.-C.; Balas, V.E.; Perescu-Popescu, L.; Mastorakis, N. Multilayer Perceptron and Neural Networks. *WSEAS Transactions on Circuits and Systems* **2009**, *8*, 579–588.
27. Tang, J.; Deng, C.; Huang, G.-B. Extreme Learning Machine for Multilayer Perceptron. *IEEE transactions on neural networks and learning systems* **2015**, *27*, 809–821.
